# Plasma MicroRNA Profiles in Rat Models of Hepatocellular Injury, Cholestasis, and Steatosis

**DOI:** 10.1371/journal.pone.0030250

**Published:** 2012-02-17

**Authors:** Yu Yamaura, Miki Nakajima, Shingo Takagi, Tatsuki Fukami, Koichi Tsuneyama, Tsuyoshi Yokoi

**Affiliations:** 1 Drug Metabolism and Toxicology, Faculty of Pharmaceutical Sciences, Kanazawa University, Kakuma-machi, Kanazawa, Japan; 2 Graduate School of Medicine and Pharmaceutical Science, University of Toyama, Sugitani, Toyama, Japan; Yonsei University College of Medicine, Republic of Korea

## Abstract

MicroRNAs (miRNAs) are small RNA molecules that function to modulate the expression of target genes, playing important roles in a wide range of physiological and pathological processes. The miRNAs in body fluids have received considerable attention as potential biomarkers of various diseases. In this study, we compared the changes of the plasma miRNA expressions by acute liver injury (hepatocellular injury or cholestasis) and chronic liver injury (steatosis, steatohepatitis and fibrosis) using rat models made by the administration of chemicals or special diets. Using miRNA array analysis, we found that the levels of a large number of miRNAs (121–317 miRNAs) were increased over 2-fold and the levels of a small number of miRNAs (6–35 miRNAs) were decreased below 0.5-fold in all models except in a model of cholestasis caused by bile duct ligation. Interestingly, the expression profiles were different between the models, and the hierarchical clustering analysis discriminated between the acute and chronic liver injuries. In addition, miRNAs whose expressions were typically changed in each type of liver injury could be specified. It is notable that, in acute liver injury models, the plasma level of miR-122, the most abundant miRNA in the liver, was more quickly and dramatically increased than the plasma aminotransferase level, reflecting the extent of hepatocellular injury. This study demonstrated that the plasma miRNA profiles could reflect the types of liver injury (e.g. acute/chronic liver injury or hepatocellular injury/cholestasis/steatosis/steatohepatitis/fibrosis) and identified the miRNAs that could be specific and sensitive biomarkers of liver injury.

## Introduction

MicroRNAs (miRNAs) are a family of short noncoding RNA whose final product is a 22-nucleotide functional RNA molecule [1]. They regulate the expression of target genes by binding to complementary regions of transcripts to repress their translation or cause mRNA degradation. There is growing evidence that miRNAs play a fundamental role in a variety of physiological and pathological processes in animals [1], [2]. At present, more than 1400, 720, and 400 miRNAs have been identified in human, mouse, and rat, respectively. Many miRNAs are expressed in a tissue or cell-specific manner. Aberrant expression of miRNAs in tissues has been implicated in a variety of diseases including cancer [3], viral hepatitis [4], and heart disease [5]. Recently, it was reported that miRNAs are present in the body fluids such as plasma [6], serum [6], urine [7], and saliva [8], [9]. Their expression patterns significantly vary in various diseases suggesting their potential as biomarkers [6], [10], [11]. The first study of a plasma miRNA profile in liver injury was from Wang et al. [12]. They comprehensively analyzed the plasma miRNA expression in mice with hepatocellular injury caused by acetaminophen (APAP) overdose. Subsequently, it was reported that the plasma miR-122 level was increased in a rat model of hepatocellular injury caused by trichlorobromomethane or carbon tetrachloride (CCl_4_) administration [13], and was increased in a mouse model of D-galactosamine/lipopolysaccharides- or alcohol-induced liver injury [14]. However, information on the plasma miRNA changes by liver injury is still limited.

Acute liver injury is classified into three types: hepatocellular injury, cholestasis, or mixed type [15], [16]. Chronic liver injury is a progressive disease showing increasing severity such as steatosis, steatohepatitis, fibrosis, cirrhosis and cancer. For the diagnosis of liver injury, alanine aminotransferase (ALT), aspartate aminotransferase (AST), alkaline phosphatase (ALP) and total bilirubin (T-Bil) in the blood are commonly used. However, these parameters cannot thoroughly identify the type of liver injury. In addition, these parameters may show increases with extrahepatic injury such as muscle damage or cardiac injury [17], [18]. Moreover, ALT is not always correlated well with the histomorphologic data of liver [19], [20]. In this study, we sought to compare the plasma miRNA expression profiles in various types of liver injury using rat models in order to evaluate whether plasma miRNAs can be markers that can distinguish the different types of liver injury. In addition, we determined the time course of changes of selected plasma miRNA levels with acute liver injury, and evaluated the utility of miRNAs as quantitative markers of liver injury.

## Materials and Methods

### Chemicals and Reagents

APAP, α-naphthyl isothiocyanate (ANIT) and CCl4 were purchased from Wako Pure Chemicals (Osaka, Japan). Methapyrilene (MP) was from Sigma-Aldrich (St. Louis, MO). Standard diets (StdD), high fat diets (HFD) and methionine choline-deficient diet (MCDD) were obtained from Oriental Yeast (Tokyo, Japan). RNAiso was from Takara (Shiga, Japan). *mir*Vana PARIS kit, Megaplex pools, TaqMan microRNA Reverse Transcription kit, TaqMan microRNA assays, TaqMan 2× Universal PCR Master Mix No AmpErase UNG and TaqMan Rodent MicroRNA Array v2.0 were from Applied Biosystems (Foster City, CA). All other chemicals and solvents were of the highest grade commercially available.

### Animal Models

Animal maintenance and treatment were conducted in accordance with the National Institutes of Health Guide for Animal Welfare of Japan, as approved by the Institutional Animal Care and Use Committee of Kanazawa University, Japan. The study was approved by the Animal Ethics Committee of Kanazawa University (No. 31203). Male 5-week-old Sprague-Dawley rats were purchased from Japan SLC (Hamamatsu, Japan). Rats were housed in a controlled environment (temperature 25±1°C, humidity 50±10%, and 12 h light/12 h dark cycle) in the institutional animal facility with access to food and water *ad libitum*. Rats were acclimatized before use for the experiments. To make the hepatocellular injury models, rats (n = 6–8) were orally administered 500 mg/kg (low dose) or 1,000 mg/kg (high dose) APAP suspended in 0.5% carboxymethylcellulose (CMC) after fasting. In some experiments, rats were administered APAP without fasting. Rats (n = 6) were orally administered 300 mg/kg MP dissolved in 0.5% CMC. These rats were sacrificed 24 h after the administration. To make cholestasis models, rats (n = 5) were orally administered 150 mg/kg ANIT dissolved in corn oil and were sacrificed 48 h after the administration. Bile duct ligated (BDL) or sham operated rats (male, 5-week-old, n = 3–4) were purchased from Japan SLC, and were sacrificed 3 days after the operations. To make steatosis and steatohepatitis models, rats (n = 5) were fed HFD and MCDD, respectively for 8 weeks. To make a fibrosis model, rats (n = 5) were intraperitoneally administered 0.5 mg/kg CCl4 dissolved in olive oil twice a week for 10 weeks, and were sacrificed 3 days after the last administration. After sacrifice, blood and liver were collected. EDTA was added as an anticoagulant to the blood and kept on ice for 30 min. After centrifugation, plasma was collected and kept at −80°C until use. A part of the liver was fixed in buffered neutral 10% formalin.

### Biochemical Assay and Pathological Examination

Plasma ALT, AST and T-Bil levels were determined by using the Dri-Chem 4000 (FUJIFILM, Saitama, Japan) according to the manufacturer's instructions. The formalin fixed samples were embedded in paraffin and sectioned, and then stained with hematoxylin-eosin for microscopic examination. A part of the frozen liver was embedded in optimal cutting temperature (O.C.T) compound (Sakura Finetek Japan, Tokyo, Japan) and sectioned, and then stained with Oil red O and hematoxylin.

### Evaluation of Stability of Plasma miRNAs

The study using human samples was approved by the Ethics Committee of Kanazawa University, Japan (No. 203). Written informed consent was obtained from all subjects. Blood samples were collected from 9 healthy male subjects (22 to 27-year-old) or 2 non-treated male rats (6-week-old). EDTA was added as an anticoagulant to the blood and kept on ice for 30 min. After centrifugation, plasma was collected and pooled. The pooled samples were divided into each 30 µL, and were kept at 4°C, room temperature or 37°C. After 3, 6, 12 and 24 h, total RNAs were prepared and individual miRNAs were measured as described below.

### TaqMan MicroRNA Assay

Total RNAs including small RNAs from the plasma were isolated using RNAiso according to the manufacturer's instructions. Dr. GenTLE Precipitation Carrier (Takara) was used as a carrier. The cDNAs were synthesized using TaqMan microRNA Reverse Transcription kit with TaqMan microRNA assays 5× reaction mix according to the manufacturer's protocol. To the cDNA sample, TaqMan 2× Universal PCR Master Mix (No AmpErase UNG) and TaqMan microRNA assays 5× reaction mix were added, and the real-time PCR was performed using MP3000P (Stratagene, La Jolla, CA) with the MxPro QPCR software.

### TaqMan MicroRNA Array Analysis

Total RNAs including small RNAs were prepared from 600 µl pooled rat plasma (n = 3–8) using the *mir*Vana PARIS kit according to the manufacturer's protocol except that the acid/phenol/chloroform extraction was repeated two times. The cDNAs were synthesized from the total RNA using Megaplex pools according to the manufacturer's protocol. Primers used in the reverse transcription reaction were TaqMan stem-loop primers for 365 individual miRNAs and 3 endogenous controls. Preamplification was performed by adding of 2× TaqMan PreAmp Master Mix and 10× Megaplex PreAmp Primers to the cDNA sample. To the preamplification products, TaqMan 2× Universal PCR Master Mix (No AmpErase UNG) were added. The entire mixture was applied to individual ports of TaqMan Array Rodent MicroRNA A+B Cards Sets v2.0, 384-well microfluidics cards containing 375 (A array) or 210 (B array) primer-probe sets for individual miRNAs. Quantitative real-time PCR was performed using a 7900HT Fast Real-Time PCR system (Applied Biosystems) with an SDS software v.2.4. Expression levels were evaluated using comparative cycle threshold (Ct) method. Ct values ranged from 0 to 40. miRNAs giving Ct values >32 in all groups were omitted from data analysis because this cut-off value was recommended by the manufacturer. The data were presented as (40 - Ct). ΔCt values [ΔCt = (40 - Ct model)−(40 - Ct control)] were calculated as fold changes. The hierarchical clustering was performed using Cluster 3.0 software (complete linkage) and mapletree.

### Statistical Analysis

Data are expressed as mean ± SD. Comparison of two groups was made with a Mann-Whitney's U-test. Comparison of multiple groups was made with Kruskal-Wallis followed by Dunn's test. A value of *P*<0.05 was considered statistically significant.

## Results

### Plasma biochemistry and histopathology of rat models of acute and chronic liver injury

**Figure 1:**
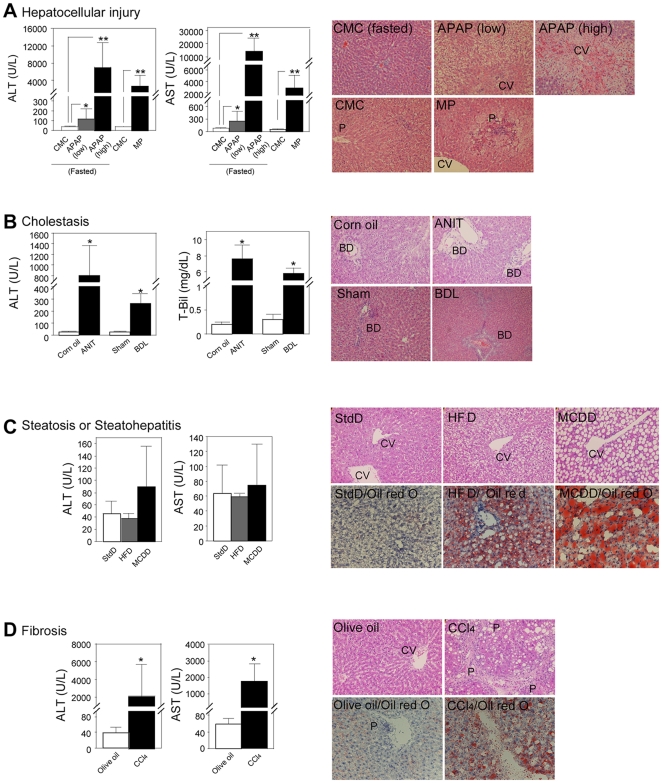
Plasma ALT, AST or T-Bil levels and histopathological changes of liver in rat models of hepatocellular injury induced by the administration of APAP (n = 6–8) or MP (n = 6) (A); cholestasis induced by the administration of ANIT, or BDL (n = 5) (B); steatosis or steatohepatitis induced by feeding of HFD (n = 5) or MCDD (n = 5) (C); and fibrosis induced by the administration of CCl4 (n = 6) (D). Data are mean ± SD. Significantly different from control group (*P<0.05 and **P<0.01). Liver sections were stained with HE for all models (original magnification×200) and Oil red O for the chronic liver injury models (original magnification×400). CV: Central vein; P: Portal region; BD: Bile duct.

The plasma ALT and AST levels were significantly elevated by the administration of APAP (Fig. 1A). The elevation of the ALT and AST levels in high dose of APAP-treated rats was considerably higher than that in low dose APAP-treated rats. The hepatocellular injury was observed at the pericentral regions. Thus, the APAP-induced hepatocellular injury model was established. Next, we sought to establish another hepatocellular injury model by the administration of MP. In the treated rats, the plasma ALT and AST levels were significantly elevated and the hepatocellular injury was observed at the periportal regions (Fig. 1A). Thus, we established two types of hepatocellular injury model. In the ANIT-treated rats, the plasma ALT and T-Bil levels were considerably elevated (Fig. 1B). Degenerating and vanishing bile ducts as well as mild necrosis or inflammation were observed. Thus, the ANIT-induced cholestasis model was established. Next, we evaluated another cholestasis model by BDL. In the treated rats, the plasma ALT and AST levels were considerably elevated (Fig. 1B). Enlargement of bile ducts as well as mild necrosis or inflammation were observed. Thus, we established two types of cholestasis model. In the HFD-fed rats, the plasma ALT and AST levels were not changed, whereas in the MCDD-fed rats, the ALT level tended to be higher than that in StdD-fed rats (Fig. 1C). Cytosolic hypertrophy and clear vacuoles containing lipid were observed in HFD-fed rats. Diffuse macrovesicular steatosis and infiltration of inflammatory cells were observed in MCDD-fed rats. Accumulation of fat was observed in both groups by Oil red O staining. Thus, we considered that the steatosis and steatohepatitis models were established. In the CCl4-treated rats, the plasma ALT and AST levels were considerably elevated (Fig. 1D). Fibrosis, adipose degeneration, and infiltration of inflammatory cells were observed at the periportal region. Accumulation of fat was also observed by Oil red O staining. Thus, we established a fibrosis model.

### Stability of plasma miRNAs

**Figure 2:**
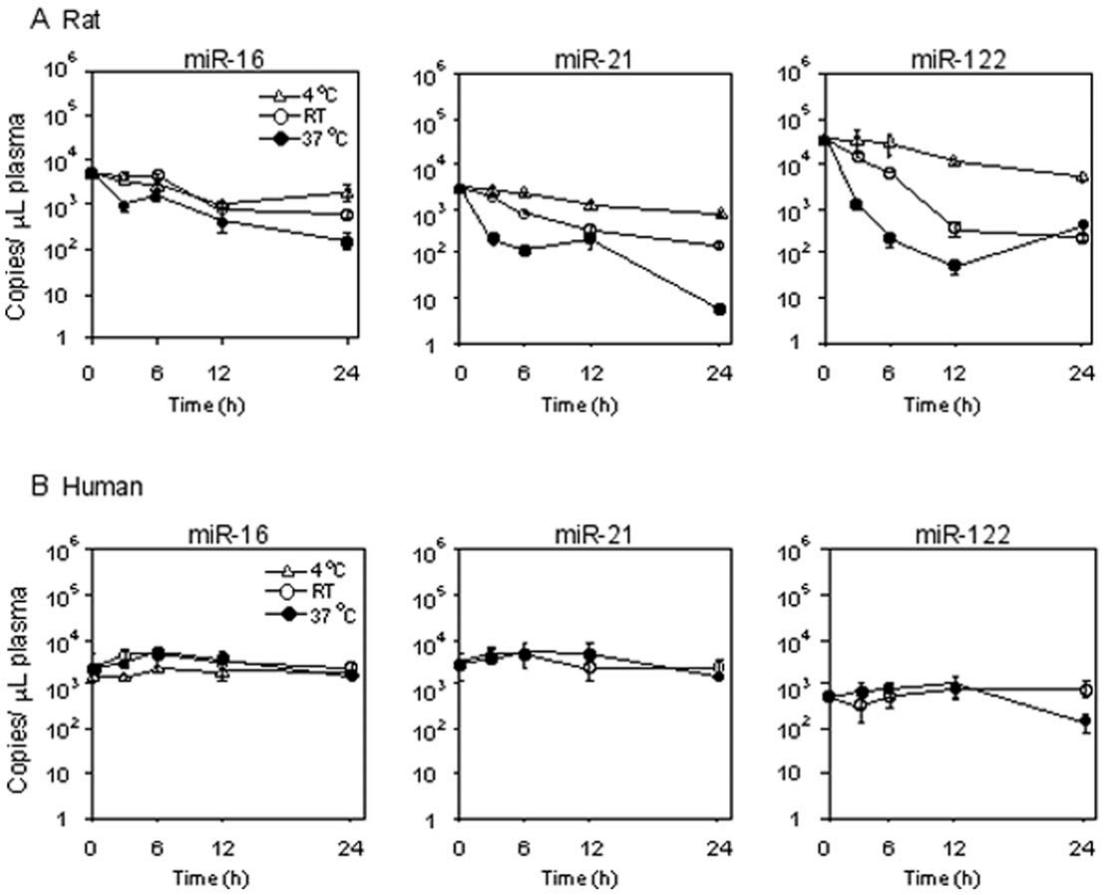
Stability of miR-16, miR-122 or miR-21 in rat (A) or human (B) plasma. Plasma samples from 2 non-treated male rats or 9 male healthy subjects were pooled and incubated at 4°C, room temperature (RT) or 37°C. Data represent copy numbers per one µL of plasma. Data are mean ± SD of triplicate determination (n = 3).

It has been reported that the miRNAs in human plasma are stable [6], but there is no information on the stability of miRNAs in rat plasma. We investigated the stability of miRNAs in rat plasma compared to those in human plasma. We chose miR-16, miR-21 and miR-122 because their sequences are identical in rat and human and these miRNAs are substantially expressed in plasma. We found that these miRNAs in rat plasma were unstable at 37°C, whereas those in human plasma were stable, supporting the previous study (Fig. 2). The extent of degradation varied depending on the kinds of rat miRNAs. The miR-21 and miR-122 were decreased to 2% and 1% of the control, respectively, after 6 h incubation at 37°C, whereas the miR-16 was sustained at 30% of the control. The level of miR-122 was 10-fold higher than the levels of miR-16 and miR-21, which showed similar levels. Thus, it appeared that the extent of degradation was not associated with the expression levels. When the plasma samples were incubated at room temperature (∼25°C), the degradation of the miRNAs was attenuated. Moreover, the degradation of miRNAs was considerably repressed when the miRNAs were kept at 4°C. Accordingly, for the subsequent study, we kept the rat plasma samples on ice until the RNA extraction.

### Plasma miRNAs expression profiles in rat models of liver injury

**Figure 3:**
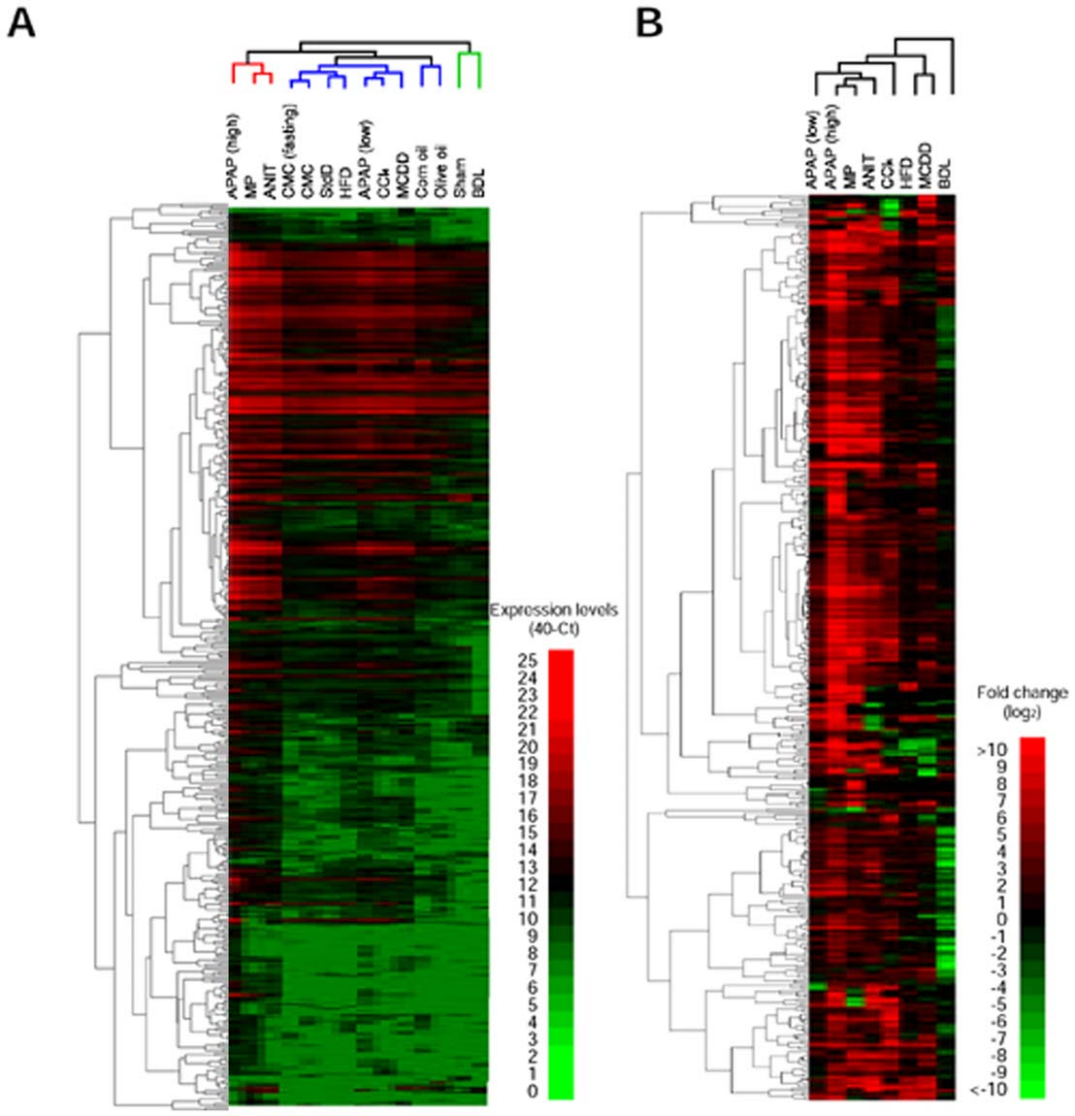
Hierarchical clustering of plasma miRNA expression profiles in rats with liver injury (A) and the fold changes between the injury model and control (B). The levels were clustered by using Cluster 3.0 software (complete linkage) and visualized by using MapleTree software. Data are presented as 40-Ct (A) and log_2_ (B) value.

The expression profiles of the plasma miRNAs in the rat models of liver injury were determined by TaqMan microRNA array analysis. The global normalization method (correction with the sum of the expression levels of detected miRNAs) is generally used for normalization in array analyses. The numbers of miRNAs that were detected, or the Ct values of which were <32, in the liver injury groups except the BDL group tended to be larger than those in the control groups (Table 1). Accordingly, we considered that the global normalization method would be inappropriate. Alternatively, we confirmed by the measurement of the cel-miR-39 levels (a miRNA in *C. elegans*) that the efficiencies of extraction and detection of miRNAs were almost equal in all groups. The number of miRNAs, which gave Ct values <32 in at least one group, was 433. We performed a clustering analysis for the expression levels of the 433 miRNA. As shown in Fig. 3A, all groups were roughly divided into three, 1) APAP (high), MP, and ANIT groups, 2) HFD, APAP (low), CCl4, MCDD groups and the controls, and 3) sham and BDL groups. Next, we examined the fold changes in the miRNA expression. The numbers of miRNAs showing >2-fold increases or <0.5-fold decreases in the liver injury groups in comparison with a control groups are shown in Table 1. In most groups, the numbers of increased miRNAs were larger than those of decreased miRNAs whereas, in the BDL group, the numbers of decreased miRNAs were larger than those of increased miRNAs. We performed clustering analysis for the fold changes of the miRNAs (Fig. 3B). It was demonstrated that the profiles of the APAP (high) and MP groups were similar. The profile of the ANIT group was similar to the above two groups, but that of the BDL group was quite different. The profiles of the CCl4, HFD, and MCDD groups were roughly close to each other, and the CCl4 group with an increased ALT level close to the groups with acute liver injury. Thus, it was demonstrated that the changes of miRNA expression were different between the acute and chronic liver injuries.

**Table 1:** Number of miRNAs whose expressions were detected and changed with liver injury in rat plasma.

Drug or diet	Type	Detectable	Ct<32	Up (>2.0-fold)	Down (<0.5-fold)
CMC (fasted)		277	181	-	-
APAP (Low)	Hepatocellular	328	231	237	18
APAP (High)	Hepatocellular	400	313	317	6
CMC		284	179	-	-
MP	Hepatocellular	381	282	295	10
Corn oil		286	177	-	-
ANIT	Cholestasis	352	259	280	10
Sham		241	137	-	-
BDL	Cholestasis	207	126	60	130
StdD		269	175	-	-
HFD	Steatosis	294	206	121	16
MCDD	Steatohepatitis	305	214	186	35
Olive oil		248	152	-	-
CCl4	Fibrosis	289	201	225	27

The total number of miRNAs on the array system is 585.

APAP: acetaminophen; MP: methapyrilene; ANIT: α-naphthyl isothiocyanate; BDL: bile duct ligation; StdD: standard diet; HFD: high fat diet; MCDD: methionine choline-deficient diet.

**Figure 4:**
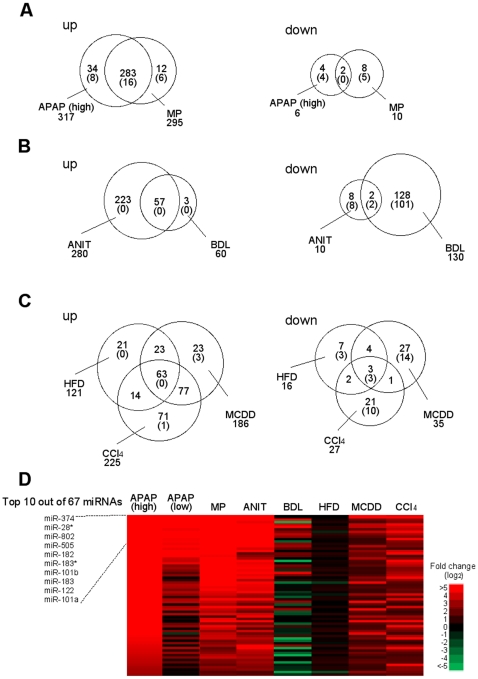
Up- or down-regulated miRNAs in hepatocellular injury models (A), cholestasis models (B), and chronic liver injury models (C). Venn diagram shows the number of changed miRNAs. The numbers in the parenthesis are the numbers of miRNAs whose expressions were specifically changed only in the given models. Heat map of 67 miRNAs in all models, which were commonly up-regulated with necrosis and inflammation (D).

Among the up-regulated miRNAs in the APAP (high) group (317 miRNAs) and MP group (295 miRNAs), 283 miRNAs were common (Fig. 4A). Among the 6 down-regulated miRNAs in the APAP (high) group and 10 down-regulated miRNAs in the MP group, only 2 miRNAs were common. Among the 280 up-regulated miRNAs in the ANIT group and 60 up-regulated miRNAs in the BDL group, 57 miRNAs were common (Fig. 4B). Among the 10 down-regulated miRNAs in the ANIT group and 130 down-regulated miRNAs in the BDL group, 2 miRNAs were common. Among the 121 up-regulated miRNAs in the HFD group, 186 up-regulated miRNAs in the MCDD group and 225 up-regulated miRNAs in the CCl4 groups, 63 miRNAs were common. Among the 16 down-regulated miRNAs in the HFD group, 35 miRNAs in the MCDD group and 27 miRNAs in the CCl4 groups, only 3 miRNAs were common. To find potential biomarkers for hepatocellular injury, cholestasis, and steatosis, the miRNAs whose expression was commonly changed in the same type of liver injury were compared across the different types of liver injury. As the results, we found that 16 miRNAs were specifically up-regulated in hepatocellular injury model, 2 miRNAs and 3 miRNAs were specifically down-regulated in cholestasis and steatosis models, respectively (Fig. 4, Table 2). It was suggested that these miRNAs would be a novel biomarker for hepatocellular injury, cholestasis, and steatosis.

**Table 2:** The miRNAs whose expressions were changed in hepatocellular injury, cholestasis and steatosis.

Type of liver injury		Fold change (log2)				Fold change (log2)		
	Up-regulated miRNAs	APAP		MP	Down-regulated miRNAs	APAP		MP
Hepatocellular injury	miR-200a*	13.2		8.7				
	let-7c-1*	10.8		3.0				
	miR-503	10.2		9.0				
	miR-337-3p	9.6		7.1				
	miR-10b	9.2		2.2				
	miR-34c	8.6		6.3	-			
	miR-327	8.5		8.8				
	miR-351	8.2		5.5				
	miR-704	7.8		9.7				
	miR-410	7.6		1.7				
	Top 10 out of 16 miRNAs							
		ANIT		BDL		ANIT		BDL
Cholestasis	-				miR-190	−5.9		−6.0
					miR-743b	−3.3		−8.3
		HFD	MCDD	CCl4		HFD	MCDD	CCl4
Steatosis	-				miR-449c	−7.0	−6.0	−2.6
					miR-410	−4.4	−4.4	−2.8
					miR-10b*	−2.5	−5.0	−5.1

Next, we looked at the miRNAs whose expression was specifically changed in each model. By the comparison across the all models, we found that 12 out of 38 miRNAs (sum of up-regulated and down-regulated miRNAs) and 11 out of 20 miRNA were specifically changed in the APAP and MP groups, respectively (Fig. 4A, Table 3). It was considered that these miRNAs could be used to know the damaged area, pericentral or periportal region. Since the 223 miRNAs and 3 miRNAs that were up-regulated in ANIT and BDL groups, respectively were also up-regulated in the hepatocellular injury models, no miRNAs were found to be specific for these models. However, 8 out of 8 miRNAs and 101 out of 208 miRNAs were specifically down-regulated in ANIT and BDL groups, respectively (Fig. 4B, Table 3). It was suggested that these miRNAs would be candidate biomarkers of intrahepatic and extrahepatic cholestasis. We found that 3 out of 28 miRNAs, 17 out of 50 miRNAs, and 11 out of 92 miRNA were specifically changed in the HFD, MCDD, and CCl_4_ groups, respectively (Fig. 4C, Table 3). It was suggested that these miRNAs would be candidate biomarkers of steatosis, steatohepatitis and fibrosis, respectively. Especially, the miRNAs whose expressions changed only in the HFD group might be associated with the fat accumulation without inflammation or fibrosis.

**Table 3:** The miRNAs whose expressions were changed only in the given model of liver injury.

Type of liver injury	Up-regulated miRNAs	Fold change (log2)	Down-regulated miRNAs	Fold change (log2)
Hepatocellular injury by APAP (high)	miR-592	10.0	miR-103	−2.7
	miR-29b-2*	8.8	miR-141*	−1.9
	miR-367	8.7	miR-764-5p	−1.9
	miR-19a*	8.7	miR-132	−1.5
	miR-344-3p	8.3		
	miR-218-1*	8.1		
	miR-10a*	8.0		
	miR-217	5.9		
Hepatocellular injury by MP	miR-697	9.6	miR-687	−9.0
	miR-200c*	8.1	miR-30a*	−8.3
	miR-879*	5.9	miR-29b	−5.8
	miR-30c-1*	4.3	miR-744*	−5.4
	miR-149	2.3	miR-181c	−4.8
	miR-29b*	1.0		
Cholestasis by ANIT	-		miR-704	−7.3
			miR-875-5p	−7.0
			miR-218-1*	−6.4
			miR-337-3p	−5.6
			miR-411	−5.0
			miR-351	−3.4
			miR-24-1*	−2.8
			miR-699	−2.6
Cholestasis by BDL	-		miR-377	−11.8
			miR-27b	−10.0
			miR-872	−9.7
			miR-130b	−9.7
			miR-185	−8.7
			miR-361	−8.1
			let-7i	−8.0
			let-7b	−7.8
			miR-99a	−7.7
			miR-17-3p	−7.6
			Top 10 out of 101 miRNAs	
Steatosis by HFD	-		miR-219-1-3p	−2.5
			miR-463	−1.1
			miR-183	−1.0
Steatohepatitis by MCDD	miR-154	9.6	miR-7a*	−10.5
	miR-503*	4.8	miR-181a	−8.6
	miR-139-3p	1.4	miR-150	−7.3
			miR-384-5p	−6.8
			miR-17*	−5.6
			miR-197	−5.4
			miR-134	−2.4
			miR-542-3p	−2.0
			miR-706	−1.8
			miR-148b-5p	−1.7
			Top 10 out of 14 miRNAs	
Fibrosis by CCl4	miR-764-5p	3.3	miR-30c-1*	−15.3
			miR-30c-2*	−12.8
			miR-302c*	−8.7
			miR-153	−7.5
			miR-9*	−6.5
			miR-503*	−5.7
			miR-376c*	−4.0
			miR-215	−1.4
			miR-30b*	−1.3
			miR-29c*	−1.2

All groups except the BDL and HFD groups showed necrosis and inflammation. Based on the fact, we sought to identify the miRNAs whose expressions were changed in response to necrosis and inflammation. By searching miRNAs whose expressions were commonly increased in the APAP, MP, ANIT, MCDD, and CCl4 groups, but not in BDL and HFD groups, we found that 67 miRNAs might reflect necrosis and inflammation (Fig. 4D). Among them, we focued on miR-122, which is the most abundant miRNA in liver [21], in the next experiment.

### Time course of plasma miRNA change in rats with acute liver injury

**Figure 5:**
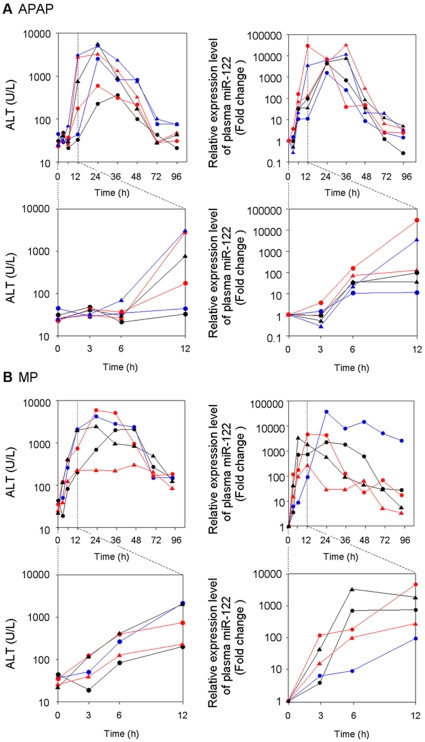
Time-dependent changes of plasma ALT and miR-122 levels in individual rat orally administered 1000 mg/kg of APAP (n = 6) with fasting (A) or 300 mg/kg MP (n = 5) (B). Graphs with magnified abscissa are also shown. The miR-122 levels represent relative value to control.

We examined the time course of the plasma miRNA changes in rats with acute liver injury, focusing on miR-122. We measured the plasma ALT and miR-122 levels over time in six rats administered 1000 mg/kg APAP. The ALT levels began to increase 6 h after the administration, reached a peak at 24 h and then decreased (Fig. 5A). In contrast, the plasma miR-122 levels began to increase 3 h after the administration, reached a peak at 12–36 h and then decreased. Although there were large interindividual differences in the ALT levels, the interindividual differences in plasma miR-122 level were relatively small. It should be noted that the change of plasma miR-122 was more dynamic than that of ALT. Next, we looked at five rats treated with 300 mg/kg MP. The ALT and miR-122 levels began to increase 3 h after the administration, but the extent and rate of the increase of miR-122 was more dramatic than those of the ALT levels (Fig. 5B). These results suggest that the plasma miRNA level changed more sensitively than the ALT level did in response to acute liver injury.

### Association of the plasma miR-122 increase with hepatocellular injury

**Figure 6:**
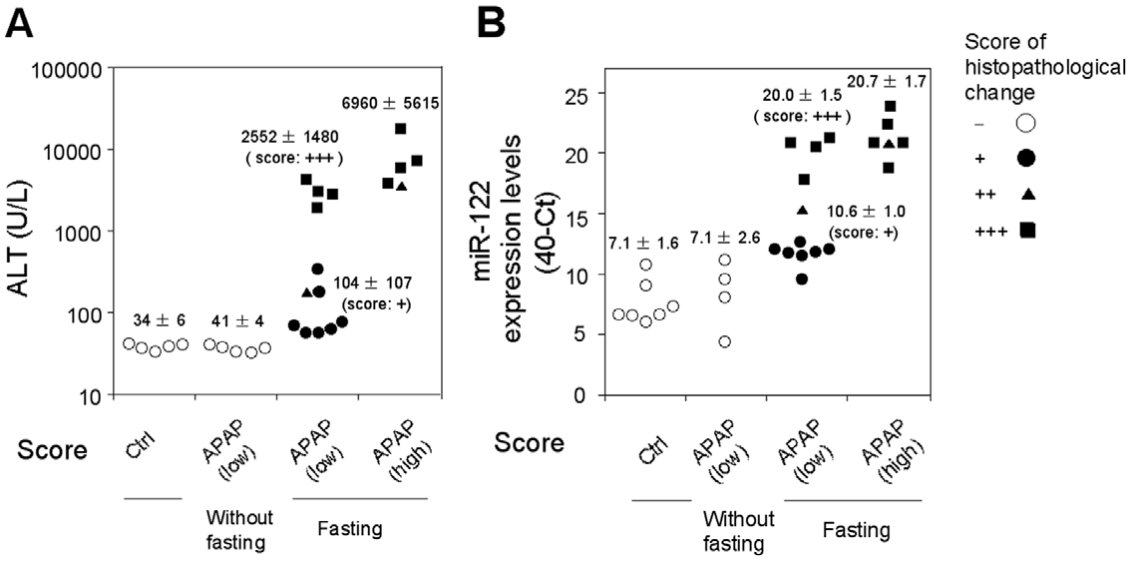
Association of plasma miR-122 level with hepatocellular injury. The extent of hepatocellular necrosis and inflammation was scored + (closed circle), ++ (closed triangle), and +++ (closed square) by histopathological examination, and was compared with the plasma ALT (A) and miR-122 (B) levels in rats administered 1000 mg/kg (high dose) or 500 mg/kg (low dose) of APAP with fasting, low dose of APAP without fasting, and CMC (as a control).

We examined whether the increase of plasma miR-122 was due to liver injury or the administered chemicals. To address this issue, 500 mg/kg of APAP were orally administered to rats without fasting. In contrast to the administration of APAP with fasting, which decreased the hepatic glutathione level, the treatment neither caused histopathological changes (data not shown) nor an elevation of ALT in rats (41±4 U/L versus 34±6 U/L in control) (Fig. 6). In these rats, the plasma miR-122 levels were almost the same as those in control rats (40-Ct value: 7.1±2.6 versus 7.1±1.6 in control). Therefore, we could conclude that the increase of the plasma miR-122 was due to liver injury, but not to APAP itself. When 500 mg/kg APAP were administered with fasting, hepatocellular necrosis and inflammation were observed in all 12 rats, with scores of + (7 rats, closed circle), ++ (1 rat, closed triangle), and +++ (4 rats, closed square). In the 7 rats showing mild (+) histopathological changes, the increase of the ALT levels was not significant (104±107, 3.1 fold of control), but the increase of the miR-122 level was remarkable (10.6±1.0, 11.3 fold of control). In addition, the extent of the increase of miR-122 mirrored the histopathological changes (++ 15.2 and +++ 20.0±1.5). In the rats administered 1000 mg/kg APAP with fasting, the interindividual variability of miRNA (20.7±1.7) was less than that of ALT (6960±5615). Thus, it is suggested that miRNA would be a more sensitive and quantitative biomarker of liver injury, with low interindividual variability, than a conventional biomarker, ALT.

## Discussion

It was reported in 2008 that miRNAs stably exist in human plasma or serum [6], [10]. After these reports, miRNAs in body fluids have been investigated in a wide variety of patient samples and animal models and have been revealed as potential biomarkers of various diseases [22]. In contrast to human miRNAs, there was no report on whether the miRNAs in rodent plasma are also stable. Therefore, we first determined the stability of miRNAs in rat plasma and found that the miRNAs in rat plasma were less stable than those in human plasma. The extent of degradation was different among the miRNAs, but was independent of the expression levels of these miRNAs. We could provide important information for dealing with rat plasma miRNAs. Although the relative instability of miRNAs in rat plasma was revealed, our finding does not hinder the potential as a biomarker, because the degradation can be suppressed if the samples are kept below 4°C or stored in freezer, and it is speculated that the circulating miRNAs in body would be stable at similar extent between human and rat, based on the fact encompassing in vesicle or binding to proteins. Thus, we consider that the miRNAs in rat plasma could also be useful as a biomarker.

In this study, we established various rat models with liver injury including hepatocellular injury (APAP or MP), cholestasis (ANIT or BDL), steatosis (HFD), steatohepatitis (MCDD), and fibrosis (CCl_4_), and determined the plasma miRNA expression profiles. In the hepatocellular injury models caused by 500 mg/kg (low dose) or 1000 mg/kg (high dose) of APAP, 231 and 313 miRNAs out of 328 and 400 detectable miRNAs were up-regulated in the plasma, respectively. In a previous study by Wang et al. [12] using a mouse model that was administered 300 mg/kg APAP, it was demonstrated that 25 miRNAs out of 53 detectable miRNAs were up-regulated (>1.5 fold). Almost all the up-regulated miRNAs (22 our of 25 miRNAs) such as miR-122, miR-192, miR-685, miR-193, and miR-29c were also up-regulated in our rat model, suggesting that common plasma miRNAs seem to be up-regulated in APAP-induced liver injury independent of species. It should be noted that the miRNAs that were detected or up-regulated in their study were considerably fewer than those in our study, although the number of probes were comparable between the studies (the hybridization-based array they used contained probes representing 576 mouse miRNAs, whereas the TaqMan real time-PCR based-array we used contained probes representing 585 rodent miRNAs). The differences may possibly be due to species differences in the expression level of miRNAs in plasma or differences in the sensitivity of the platforms used for array analysis.

Wang et al. [12] also reported using a mouse model with APAP that the levels of the miRNAs, which were increased in plasma, were decreased in liver. They described that the cellular damage in the liver tissue resulted in the transport or release of cellular miRNAs into the plasma, which may be a similar process by which cellular enzymes are released after cellular damage. Although we have not confirmed yet whether such an inverse correlation also exists in rat models, it might be true because the miRNAs whose expressions in plasma were increased were tended to be common in the models representing hepatocellular damage (APAP, MP, ANIT, MCDD, and CCl_4_ groups). Additionally, we found miRNAs whose expressions were specifically changed in the APAP and MP group. APAP and MP induced hepatocellular necrosis at the pericentral and periportal region, respectively. Thus, such specific miRNAs would be useful to determine the damaged area. A likely explanation for the finding that different miRNAs were altered in plasma between the APAP and MP groups might be that the miRNAs which are highly expressed at the pericentral region may be selectively released to plasma in APAP group and the miRNAs which are highly expressed at the periportral region may be selectively released to plasma in MP group, although it remains to be clarified whether the miRNAs in liver may be differently expressed at pericentral and periportal regions. It would be quite possible that the miRNA expression profiles of the pericentral and periportal regions are different, because it is well known that some proteins show zonal expression, which would be due to differences in transcriptional regulation [23], [24]. We now determine the expression of miRNAs in liver at different zones, and will investigate the relationship between the changes of miRNA in the liver and plasma in rat models of liver injury in the future.

We found that the changes in plasma miRNA in the hepatocellular injury models were more dynamic than those in ALT (Fig. 5). One of the reasons would be the difference in the type of detection (real-time RT-PCR for miRNAs versus colorimetric assay for ALT activity). In addition, it has been reported that ALT is mainly expressed in the portal vein area [19], whereas our preliminarily study revealed that the miR-122 uniformly shows high expression in liver at the pericentral and periportal regions (unpublished data). That might be another reason for the more sensitive response of miR-122 than ALT toward the liver injury which would be a benefit as a biomarker. Moreover, we showed that the plasma miR-122 level was quantitatively correlated with the extent of histopathologic changes (Fig. 6). Thus, as represented by miR-122, the profile of miRNA expression could serve as a tool for understanding the onset and progression of liver injury.

In this study, ANIT-administration or BDL was used to make the cholestasis model. ANIT causes intrahepatic cholestasis by damaging the cholangiocytes lining the bile ducts [25], whereas BDL causes extrahepatic cholestasis by blocking the drainage of bile from the liver to the duodenum. As shown in Fig. 3 and Table 1, the plasma miRNA profiles in the two models were quite different, which may be due to differences in the mechanisms causing cholestasis. Since the up-regulated miRNAs were almost common with those in the hepatocellular injury model as described above, these miRNAs cannot be biomarkers of cholestasis. Instead, we could identify the miRNAs that can be biomarkers of cholestasis, among the down-regulated miRNAs. Why was such a large number of miRNAs decreased in plasma of BDL group? One may consider that the miRNAs may be instable in plasma with high levels of bile acids. However, the possibility may be denied because a recent study reported that miRNAs are present in bile that is abundant in bile acids and bilirubin [26]. Another possibility is that the bile acids accumulated in hepatocytes inhibit the secretion of miRNAs. To obtain the answer, the determination of hepatic miRNA expression profiles in ANIT and BDL groups might be useful.

In the chronic liver injury models including the HFD, MCDD, and CCl_4_ groups, we found that 3 miRNAs (miR-10b*, miR-410, miR-499) were commonly down-regulated, but were not affected in the acute liver injury models (Fig. 4), suggesting that these miRNAs might serve as biomarkers of steatosis. In addition, we found miRNAs whose expressions were specifically modulated in each model of chronic liver injury (Table 2), which might represent markers of each pathology. Previously, Jin et al. determined the miRNA expression profiles in liver from an HFD-induced steatosis model rat and found that miR-132 and miR-30d were up-regulated in liver [27]. In our corresponding model, the miR-132 was up-regulated, whereas the miR-30d was down-regulated in plasma. The miRNA expression profle in liver from MCDD-induced steatohepatitis mice model has been determined by two research groups. Dolganiuc et al. reported that 10 and 2 miRNAs were up- and down-regulated, respectively [28]. Pogribny et al. reported that each of 4 miRNAs were up- and down-regulated [29]. The only common miRNA in these studies was miR-200b, which showed up-regulation. We looked at the expression changes in plasma for these miRNAs in our rat model of steatohepatitis by MCDD. Among 13 up-regulated miRNAs in liver, 7 miRNAs including miR-200b were up-regulated, but 4 miRNAs were not changed in plasma (Two miRNAs were absent in the array platform we used). Among the 6 down-regulated miRNAs in liver, in plasma 4 miRNAs were up-regulated, but 2 miRNAs were not changed. Recently, Li et al. reported that 16 miRNAs including miR-34, miR-199a-5p, miR-221, miR-146b, and miR-214 showed progressive up-regulation in rat with hepatic fibrosis caused by dimethylnitrosamine [30]. Murakami et al. reported that 11 miRNAs including miR-34, miR-199a-5p, miR-199, miR-200, and let-7e were up-regulated in a CCl_4_-induced fibrosis model mouse [31]. Among them, miR-34 and miR-199a-5p were common in the two models. In our CCl_4_-induced fibrosis model, the miR-34a in plasma was increased, whereas the miR-199a-5p in plasma was not changed. Taken together, it seems that there is no settled rule for the relationship between the changes of miRNA in liver and in plasma. In addition to the hypothesis from the acute liver injury model that miRNAs would be released from liver to plasma, other mechanisms may also be involved in the chronic liver injury. That might explain why the change of miRNA expression in liver is not necessarily associated with that in plasma. It has been recognized that circulating miRNAs are released from cells in membrane-bound vesicles such as exosomes or microvesicles. However, recent studies reported that a significant fraction of the extracellular miRNAs is not within the vesicles, being associated with proteins [32]. It seems that there are three types of miRNAs: miRNAs which are dominantly vesicle-associated, those which are dominantly associated with protein, and those which are equally distributed. In addition, the mechanisms by which miRNAs are taken up by cells are not fully understood. To understand the relationship between the miRNA expression profiles in plasma and those in liver, the complex export and import systems of the miRNAs in various organs should be clarified.

By the comparison of the miRNA expression profiles in rat models of various types of liver injury, we could identify miRNAs that could be specific and sensitive biomarkers of hepatocellular injury, cholestasis, steatosis, steatohepatitis, and fibrosis. It is conceivable that the plasma miRNAs would be a superior noninvasive biomarker in human that could distinguish the different types of liver injury to conventional biomarkers such as ALT and ALP, although the analysis to compare the plasma miRNA expression profiles in patients suffering from various type liver injury remains to be performed. The plasma miRNAs have a potential to be used to know the types of liver injury to decide appropriate therapy, or to know the progress or restoration of liver injury in clinics. In addition, the plasma miRNAs would be useful in drug development, since they could detect liver injury caused by treatment with drug candidates at the early stage, resulting in saving time and resources in nonclinical study.

In conclusion, the present study demonstrated that the expression profiles of plasma miRNAs differed according to the type of liver injury. Although earlier studies reported the changes of some miRNAs in plasma or tissues with disease using a single model, implying the possibility of associations with the development of disease, comparison of the miRNA expression profiles across models would be important for understanding the physiological implications of the miRNAs changes. We could identify miRNAs which could be specific and sensitive biomarkers of each type of liver injury (e.g. acute/chronic liver injury or hepatocellular injury/cholestasis/steatosis/steatohepatitis/fibrosis) using rat models. Further studies are warranted to elucidate whether the miRNAs could be used as biomarkers in patients with various types of liver injury.
